# Estimation of Genetic Parameters of Type Traits in First Parity Cows of the Autochthonous Cika Cattle in Slovenia

**DOI:** 10.3389/fgene.2021.724058

**Published:** 2021-11-22

**Authors:** Mojca Simčič, Barbara Luštrek, Miran Štepec, Betka Logar, Klemen Potočnik

**Affiliations:** ^1^ Department of Animal Science, Biotechnical Faculty, University of Ljubljana, Ljubljana, Slovenia; ^2^ Agricultural Institute of Slovenia, Ljubljana, Slovenia

**Keywords:** Cika cattle, first parity cow, type traits, genetic parameters, variance components

## Abstract

The aim of this study was to estimate genetic parameters of 26 individual and four composite type traits in first parity Cika cows. An analysis of variance was performed with the generalized linear model procedure of the SAS/STAT statistical package, where the fixed effects of year of recording, cow’s age at recording and days after calving as a linear regression were included in the model. The variance components for the direct additive genetic effect and the herd effect in all type traits were estimated using the REML method in the VCE-6 software package. The estimated heritabilities ranged from 0.42 to 0.67 for the measured body frame traits, from 0.36 to 0.80 for the scored autochthonous traits, from 0.11 to 0.61 for the scored body frame traits, and from 0.20 to 0.47 for the scored udder traits. The estimated heritabilities for the composite traits called “autochthonous characteristics”, “muscularity”, “body frame” and “udder” were 0.55, 0.19, 0.19, and 0.26, respectively. The estimated genetic correlations among the measured body frame traits were positive and high, while the majority of them among the scored body frame traits were low to moderate. The estimated proportions of variance explained by the herd effect for the composite traits “autochthonous characteristics,” “muscularity,” “body frame” and “udder” were 0.09, 0.28, 0.14, and 0.10, respectively. The estimated heritabilities for the type traits of first parity Cika cows were similar to those reported for other breeds where breeding values have been routinely predicted for a long time. All estimated genetic parameters are already used for breeding value prediction in the Cika cattle population.

## Introduction

Cika cattle is a Slovenian autochthonous dual-purpose breed. The breeding goal is to preserve the original type traits of the breed and to prevent an increase of inbreeding. Cika cattle are widespread all over Slovenia, especially in their regions of origin (Bohinj, Kamnik, Tolmin). The coat color pattern is red pied sided, which is typical for this breed and very different from other cattle breeds in Slovenia. Some Cika animals have a coat color pattern similar to Pinzgauer cattle and some to Tux-Zillertaler cattle from Austria (Sambraus, 1999). Based on microsatellite genotyping, Cika cattle form an Eastern Alpine breed cluster with Pinzgauer and Pustertaler cattle. Cika cattle are considered as an authentic and valuable genetic resource (Felius et al., 2011; Simčič et al., 2013). The possibility of excluding admixed animals (sires and sire dams) from the Cika cattle breeding program using SNP haplotypes has been reported in detail elsewhere (Simčič et al., 2015a,b).

Cika cattle breeding is based on the recording of type traits of all first parity cows (Žan Lotrič et al., 2010). The age of first parity cows at the recording day is restricted to a minimum of 560 days and there are no restrictions on the maximum age. Recording time is adjusted to the rearing technology, so the animals are not recorded during the grazing season. According to the breeding program, the recording of first parity cows is planned to take place 15–120 days after calving, which is often not possible due to the grazing season in the higher mountain pastures.

The current population size of Cika cattle is 5,531 animals (www.fao.org/dad-is), which is lower than in all other breeds in Slovenia. Therefore, all breeding animals are scored by a single expert. Compared to the small population size, a large number of sires (98 in the year 2020) is used for natural service and artificial insemination in the population. The effective population size is estimated to be 117 animals. Of the animals born in 2016, 96.6% had complete pedigree data in the second generation, while 65.7% of the animals had a complete pedigree in the sixth generation. The inbreeding coefficient in the population is estimated to be 2.1%.

Genetic parameters for type traits in dairy cattle breeds have been reported in first parity cows of Holstein-Friesian (Van Dorp et al., 1998; Rupp and Boichard, 1999; Larroque and Ducrocq, 2001; Neuenschwander et al., 2005; de Haas et al., 2007; Němcová et al., 2011), Jersey (Thomas et al., 1985; Norman et al., 1988; Rogers et al., 1991), Brown Swiss (Norman et al., 1988; Dal Zotto et al., 2007; de Haas et al., 2007; Samore et al., 2010), Red Holstein (de Haas et al., 2007), Ayrshire, Guernsey and Shorthorn (Norman et al., 1988), where correlations among type traits, milk yield, fertility and longevity were estimated.

In first parity cows of beef breeds, genetic parameters for type traits were studied in Belgian blue cattle with double muscling by Gengler et al. (1995), and in Spanish Asturiana de los Valles cattle by Guitiérrez and Goyache (2002), and Guitiérrez et al. (2002). Forabosco et al. (2004) indirectly estimated longevity using correlated type traits. Genetic parameters for type traits in first parity cows of some autochthonous cattle breeds were reported as well, e.g. in the Italian autochthonous breeds Chianina (Forabosco et al., 2004), Piemontese (Mantovani et al., 2010), Rendena (Mazza and Mantovani, 2012; Mazza et al., 2014), Cabanina (Comunod et al., 2013), Valdostana cattle (Mazza et al., 2015), Alpine Grey cattle (Mancin et al., 2021) and Italian Simmental (Frigo et al., 2013).

The aim of this pilot study was to estimate genetic parameters for the measured and scored type traits included in the routine breeding values estimation of Cika cows according to the breeding program.

## Materials and Methods

### Data

The data were obtained from the Central database for cattle kept by the Agricultural Institute in Slovenia, which included information from the type traits recording of Slovenian first parity Cika cows scored in the years from 2006 to 2019. We assumed that the type trait recording was intended for first parity cows, so we limited the age at recording to a maximum of 1,460 days or 4 years as recommended by de Haas et al. (2007) and a minimum of 607 days. On average, the animals were 1,030.7 ± 167.3 days old at recording. In addition, we excluded all cows younger than 4 years of age that were scored after the second parity. First parity cows that were not in the recommended stage of lactation on the day of recording according to the breeding program were not excluded from further analysis. Consequently, the recorded first parity cows were one to 446 days after calving, 227.5 ± 112.7 days on average. After applying these limitations, 1,815 first parity cows were included in the final analysis (Table 1).

**TABLE 1:** Descriptive statistics of analyzed traits.

Traits	n	Mean ± SD	Min	Max	Descriptor^a^
Measured body frame traits					
Wither height (cm)	1,815	125.18 ± 5.28	108.0	142.0	—
Rump height (cm)	1,815	128.50 ± 5.37	113.0	146.0	—
Body length (cm)	1,815	125.66 ± 6.42	105.0	162.0	—
Chest girth (cm)	1,812	173.88 ± 9.64	150.0	208.0	—
Scored autochthonous traits					
Head length	1,815	5.45 ± 1.26	2	9	long - short
Head nobility	1,179	5.66 ± 1.15	2	9	heavy - fine
Eyes	1,815	5.71 ± 1.05	2	9	small – large
Horn base circumference	1,765	5.02 ± 1.35	1	9	thick – thin
Horns length	1,571	5.56 ± 1.33	2	9	long - short
Horns direction	1,765	5.37 ± 1.60	2	9	outward - forward
Neck	1,815	5.51 ± 1.28	2	9	heavy - fine
Dewlap	1,811	5.25 ± 1.39	2	9	heavy - fine
Coat color	1,815	5.14 ± 1.21	1	9	very dark - very bright
Back stripe	1,815	5.21 ± 1.42	1	9	wide - narrow
Rear legs stripe	1,811	5.40 ± 1.68	1	9	wide - narrow
Front legs stripe	1,812	6.25 ± 1.56	1	9	wide - narrow
Scored body frame traits					
Top line	1,815	4.76 ± 0.65	2	7	weak - straight
Rump angle	1,815	5.24 ± 0.84	2	8	high pins – extreme sloped
Rear leg set	1,815	5.55 ± 0.77	3	8	straight - sickled
Hock quality	1,815	5.74 ± 1.28	2	9	a lot of fluid – clean and dry
Foot angle	1,815	5.57 ± 1.05	2	8	low - steep
Heel height	1,815	5.49 ± 1.03	2	9	low - tall
Scored udder traits					
Fore udder attachment	1,815	4.95 ± 1.09	2	8	loose - strong
Udder depth	1,815	5.78 ± 1.10	2	8	deep - shallow
Teat thickness	1,815	4.91 ± 1.07	2	9	thin - thick
Front teat length	1,814	5.33 ± 1.16	2	9	short - long
Composite traits					
Autochthonous characteristics	1,815	5.44 ± 1.47	1	9	poor - excellent
Muscularity	1,815	5.36 ± 1.13	2	9	poor - excellent
Body frame	1,815	5.57 ± 1.14	2	8	fine - heavy
Udder	1,815	4.98 ± 1.14	1	8	poor - excellent

a Minimum = 1, maximum = 9

All first parity cows (1,815) with type traits and their known ancestors were included in the additive relationship matrix among all animals (2,953 animals in total). Almost 98% of the first parity cows had a known sire and 96% had a known dam. In total, 95% of the first parity cows had both parents known.

### Estimation of Variance Components

To investigate the non-genetic effects to be included in the model, an analysis was performed using the GLM (generalized linear model) procedure in the SAS/STAT statistical package (version 9.4, SAS Institute Inc, 2001) with the statistical model

$$y_{ijk}=\;\mu +Y_{i}+b_{I}\left(x_{ijk}-\bar{x}\right)+b_{II}\left(z_{ijk}-\bar{z}\right)+e_{ijk}$$

where *y*_*ijk*_ was the type trait, *μ* was the mean of the population, *Y*_*i*_ was the fixed effect of the recording year (i = 2006, … , 2019), *b*_*I*_ was the linear regression coefficient for the age at recording, *x*_*ijk*_ was the age at recording (days), $\bar{x}$ was the mean age at recording, *b*_*II*_ was the linear regression coefficient for days after calving, *z*_*ijk*_ was the number of days after calving, $\bar{z}$ was the mean number of days after calving and *e*_*ijk*_ was an error.

The matrix notation for the complete model including both non-genetic and genetic effects was expressed as:

y=Xβ+Wq+Zu+e

Where y is an N x 1 vector of observations, β is the vector of systematic fixed effects of order p, q is the vector of herd effect considered as random effect, u is the vector of animal effects with order m, and e is the vector of residual effects. Likewise, X, W and Z are the corresponding incidence matrices with the appropriate dimensions.

The variance components and heritability for each individual and composite trait were estimated with the REML method in the VCE-6 software package (Groeneveld et al., 2010).

The analyzed type traits were divided into six groups according to specific body regions (Simčič et al., 2016). The measured body frame traits (wither height, rump height, body length, chest girth) were assigned to Group 1. The scored autochthonous traits were divided into three groups: head characteristics (head length, head nobility, eyes, horn base circumference, horns length, horns direction) were assigned to Group 2, neck characteristics (neck, dewlap) were assigned to Group 3, and coat color traits (coat color, back stripe, rear legs stripe, front legs stripe) were assigned to Group 4. The scored body frame traits (top line, rump angle, rear leg set, hock quality, foot angle, heel height, body frame as a composite trait) were assigned to Group 5. Udder traits (fore udder attachment, udder depth, teat thickness, front teat length, udder as a composite trait) were assigned to Group 6. The variance components and genetic parameters for these anatomically similar traits divided into six groups were estimated separately for each group using multivariate mixed models. The two remaining composite traits (autochthonous characteristics, muscularity) were analyzed with a univariate mixed model.

## Results and Discussion

### Variance Component Estimates

The heritabilities (h^2^) for all included type traits in the first parity Cika cows ranged between 0.11 and 0.80, while the proportions of variance explained by the herd effect ranged between 0.01 and 0.28. Considering the six different trait groups, the heritabilities for the measured body frame traits ranged from 0.42 to 0.67, for the scored autochthonous traits from 0.36 to 0.80, for the scored body frame traits from 0.11 to 0.61, and for the scored udder traits from 0.20 to 0.47. The estimated heritabilities for the composite traits of autochthonous characteristics, muscularity, body frame, and udder were 0.55, 0.19, 0.19, and 0.26, respectively (Table 2).

**TABLE 2:** Estimated proportions of variance components ± standard errors for type traits in the first parity Cika cows.

Traits	h^2^	c^2^	e^2^
Measured body frame traits			
Wither height	0.66 ± 0.05	0.10 ± 0.02	0.24 ± 0.04
Rump height	0.67 ± 0.05	0.11 ± 0.02	0.22 ± 0.04
Body length	0.54 ± 0.05	0.13 ± 0.02	0.33 ± 0.04
Chest girth	0.42 ± 0.05	0.25 ± 0.03	0.33 ± 0.04
Scored autochthonous traits			
Head length	0.48 ± 0.03	0.06 ± 0.01	0.46 ± 0.03
Head nobility	0.43 ± 0.04	0.08 ± 0.02	0.49 ± 0.04
Eyes	0.39 ± 0.03	0.06 ± 0.02	0.55 ± 0.03
Horn base circumference	0.52 ± 0.03	0.11 ± 0.02	0.37 ± 0.03
Horns length	0.53 ± 0.03	0.04 ± 0.01	0.43 ± 0.03
Horns direction	0.36 ± 0.03	0.05 ± 0.02	0.60 ± 0.03
Neck	0.47 ± 0.04	0.07 ± 0.02	0.47 ± 0.05
Dewlap	0.63 ± 0.04	0.05 ± 0.02	0.32 ± 0.04
Coat color	0.63 ± 0.04	0.05 ± 0.01	0.32 ± 0.04
Back stripe	0.80 ± 0.04	0.02 ± 0.01	0.18 ± 0.04
Rear legs stripe	0.78 ± 0.04	0.02 ± 0.009	0.20 ± 0.04
Front legs stripe	0.67 ± 0.04	0.01 ± 0.007	0.32 ± 0.04
Scored body frame traits			
Top line	0.19 ± 0.05	0.09 ± 0.02	0.72 ± 0.05
Rump angle	0.32 ± 0.03	0.01 ± 0.008	0.67 ± 0.04
Rear leg set	0.15 ± 0.03	0.11 ± 0.02	0.74 ± 0.03
Hock quality	0.61 ± 0.04	0.05 ± 0.01	0.34 ± 0.04
Foot angle	0.11 ± 0.02	0.16 ± 0.02	0.73 ± 0.03
Heel height	0.12 ± 0.02	0.23 ± 0.02	0.65 ± 0.03
Scored udder traits			
Fore udder attachment	0.20 ± 0.03	0.13 ± 0.02	0.66 ± 0.03
Udder depth	0.26 ± 0.03	0.13 ± 0.02	0.61 ± 0.04
Teat thickness	0.34 ± 0.03	0.10 ± 0.02	0.56 ± 0.03
Front teat length	0.47 ± 0.05	0.06 ± 0.01	0.47 ± 0.04
Composite traits			
Autochthonous characteristics	0.55 ± 0.05	0.09 ± 0.02	0.36 ± 0.04
Muscularity	0.19 ± 0.05	0.28 ± 0.03	0.53 ± 0.04
Body frame	0.19 ± 0.03	0.14 ± 0.02	0.67 ± 0.03
Udder	0.26 ± 0.03	0.10 ± 0.02	0.64 ± 0.03

h^2^ = heritability, c^2^ = herd effect, e^2^ = residual.

The estimated h^2^ for measured wither height and rump height in the first parity Cika cows were very similar, 0.66 ± 0.05 and 0.67 ± 0.05, respectively (Table 2). A lower h^2^ for wither height was estimated in first parity Piemontese cows (0.31 ± 0.02; Mantovani et al., 2010). A similar h^2^ for rump height was estimated in first parity Holstein cows (0.69 ± 0.03), American Brown Swiss cows (0.64 ± 0.02), and Red Holstein cows (0.74 ± 0.03) in Switzerland (de Haas et al., 2007), whereas the estimated h^2^ was lower in first parity Brown Swiss cows in Slovenia (0.46; Špehar et al., 2012). A lower h^2^ for rump height was estimated in first parity Rendena cows (0.52; Mazza et al., 2014), American Brown Swiss cows (0.32; Dal Zotto et al., 2007), and first parity Holstein-Friesian cows in the Czech Republic (0.45; Němcová et al., 2011). The estimated h^2^ for body length in the first parity Cika cows was 0.54 ± 0.05 (Table 2). A lower h^2^ for body length was estimated in Rendena cows (0.41; Mazza et al., 2014). The estimated h^2^ for chest girth in first parity Cika cows was 0.42 ± 0.05, which was only slightly higher than in Holstein (0.38 ± 0.02), American Brown Swiss (0.35 ± 0.02) and Red Holstein cows (0.36 ± 0.02) in Switzerland (de Haas et al., 2007).

The estimated h^2^ for head length in the first parity Cika cows was 0.48 ± 0.03 (Table 2). A lower h^2^ for head length was estimated in the Spanish Asturiana de los Valles (0.25 ± 0.02; Gutiérrez and Goyache, 2002), and in Piemontese cows (0.15 ± 0.02; Mantovani et al., 2010). The estimated h^2^ for the composite autochthonous trait in first parity Cika cows was 0.55 ± 0.05 (Table 2). In Asturiana de los Valles cows, the estimated h^2^ for a similar composite trait called “breed characteristics” was 0.33 ± 0.02 (Gutiérrez and Goyache, 2002). The estimated h^2^ for the coat color and coat pattern traits (coat color, back stripe, rear legs stripe, front legs stripe) in the first parity Cika cows were between 0.63 and 0.80.

The estimated h^2^ for the top line in the first parity Cika cows was 0.19 ± 0.05 (Table 2). A lower h^2^ for top line was estimated in first parity Asturiana de los Valles cows (0.11 ± 0.01; Gutiérrez and Goyache, 2002), Piemontese cows (0.07 ± 0.01; Mantovani et al., 2010), Brown Swiss cows in Slovenia (0.16; Špehar et al., 2012) and American Brown Swiss cows (0.10; Dal Zotto et al., 2007). Estimated h^2^ for rump angle in first parity Cika cows was 0.32 ± 0.03 (Table 2). A similar h^2^ for rump angle was estimated in first parity Rendena cows (0.36; Mazza et al., 2014) and Czech Holstein-Friesian cows (0.34; Němcová et al., 2011). The h^2^ for rump angle in first parity Brown Swiss cows in Slovenia was 0.22 (Špehar et al., 2012), and 0.24 in American Brown Swiss cows (Dal Zotto et al., 2007). The estimated h^2^ for rear leg set in the first parity Cika cows was 0.15 ± 0.03 (Table 2). Similar h^2^ for rear legs set was found in the Rendena cows (0.21; Mazza et al., 2014), Piemontese (0.12 ± 0.02; Mantovani et al., 2010), Slovenian Brown Swiss (0.13; Špehar et al., 2012), American Brown Swiss cattle (0.14; Dal Zotto et al., 2007) and Czech Holstein-Friesian cows (0.16; Němcová et al., 2011). The estimated h^2^ for hock quality in the first parity Cika cows was 0.61 ± 0.04 (Table 2). A lower h^2^ for hock quality was estimated in Slovenian Brown Swiss (0.11; Špehar et al., 2012) and American Brown Swiss cows (0.08; Dal Zotto et al., 2007). The estimated h^2^ for heel height in first parity Cika cows was 0.12 ± 0.02 (Table 2). An equal or very similar h^2^ for heel height was estimated in Rendena cows (0.12; Mazza et al., 2014), Piemontese (0.09 ± 0.01; Mantovani et al., 2010), Slovenian Brown Swiss (0.04; Špehar et al., 2012), American Brown Swiss (0.09; Dal Zotto et al., 2007) and in Czech Holstein-Friesian cows (0.10; Němcová et al., 2011). Likewise, the estimated h^2^ for the composite trait body frame in the first parity Cika cows was 0.19 ± 0.03, which was very similar to Rendena cows (0.18; Mazza et al., 2014), an autochthonous small frame cattle breed from north-eastern Italy.

The estimated h^2^ for udder attachment in the first parity Cika cows was 0.20 ± 0.03 (Table 2), which was lower than in Rendena cows (0.32; Mazza et al., 2014), and similar to Czech Holstein-Friesian (0.24; Němcová et al., 2011) as well as French Holstein cows (0.18; Rupp and Boichard, 1999). The estimated h^2^ for fore udder attachment was higher than in Slovenian Brown Swiss (0.14; Špehar et al., 2012) and American Brown Swiss cows (0.14; Dal Zotto et al., 2007). The estimated h^2^ for udder depth in the first parity Cika cows was 0.26 ± 0.03, similar to Rendena cows (0.27; Mazza et al., 2014), Slovenian Brown Swiss (0.22; Špehar et al., 2012), American Brown Swiss (0.23; Dal Zotto et al., 2007), Czech Holstein-Friesian (0.32; Němcová et al., 2011) and French Holstein cows (0.29; Rupp and Boichard, 1999). The estimated h^2^ for teat thickness in first parity Cika cows (0.34 ± 0.03; Table 2) was similar to Rendena (0.34; Mazza et al., 2014), Slovenian Brown Swiss (0.33; Špehar et al., 2012), American Brown Swiss (0.32; Dal Zotto et al., 2007), Czech Holstein-Friesian (0.28; Němcová et al., 2011) and French Holstein cows (0.30; Rupp and Boichard, 1999). Taking into account estimated composite trait “udder” in the first parity Cika cows (h^2^ = 0.26 ± 0.03; Table 2), the results were lower than in Rendena cows (0.37; Mazza et al., 2014), and higher than in Slovenian Brown Swiss cows (0.16; Špehar et al., 2012).

The estimated h^2^ for muscularity in the first parity Cika cows was 0.19 ± 0.05 (Table 3), which was similar to Asturiana de los Valles cows (0.22 ± 0.01; Gutiérrez and Goyache, 2002) and Brown Swiss cows (0.16; Špehar et al., 2012). On the other hand, h^2^ for muscularity in the first parity Cika cows was lower than in Rendena (0.31; Mazza et al., 2014), American Brown Swiss (0.42 ± 0.02) and Red Holstein cows (0.59 ± 0.03) (de Haas et al., 2007). The huge variability in the heritabilities for the trait muscularity could be due to the recording procedure, the degree of harmonization among the experts, the statistical models, and data quality.

**TABLE 3:** Estimated genetic correlations ± standard errors (above diagonal) and phenotypic correlations (below diagonal) for measured body frame traits from Group 1.

Trait	Wither height	Rump height	Body length	Chest girth
Wither height	—	0.99 ± 0.002	0.98 ± 0.009	0.91 ± 0.02
Rump height	0.98	—	0.97 ± 0.01	0.90 ± 0.03
Body length	0.83	0.83	—	0.94 ± 0.02
Chest girth	0.70	0.70	0.75	—

The proportion of variance in the type traits of the first parity Cika cows explained by the effect of the herd (c^2^) ranged from 0.01 to 0.28 (Table 3). Interestingly, c^2^ was on average higher for measured body frame traits (0.10–0.25) compared to the scored body frame traits (0.01–0.23). Likewise, the scored autochthonous and udder traits had a low c2 (0.01–0.11 and 0.06 to 0.13, respectively). The estimated c^2^ for the composite traits (autochthonous characteristics, muscularity, body frame, udder) were 0.09, 0.28, 0.14, and 0.10, respectively. This might reflect the effect of the huge variability of production systems on the farms that cause variability in the body condition of the animals in the herd. The proportion of residual variance ranged from 0.18 to 0.74. The standard errors of the variance components varied between 0.007 and 0.05.

### Genetic and Phenotypic Correlations

The estimated genetic and phenotypic correlations between the measured body frame traits (Table 3) were positive and very high. Based on these high correlations, it would be recommended to reduce the number of measured traits in the scoring form, to make the whole procedure less time consuming. The ICAR guidelines for conformation recording (ICAR, 2018) recommend only rump height (called “stature”) to be scored. Likewise, Mazza et al. (2014) found high genetic (0.79) and phenotypic (above 0.53) correlations between body frame traits in first parity Rendena cows. On the other hand, lower positive genetic correlations between rump height and chest girth were estimated in Holstein (0.45), American Brown Swiss (0.34) and Red Holstein cows (0.54) (de Haas et al., 2007).

The estimated genetic correlations between the traits describing parts of the head (Table 4) were low to moderate, and mostly positive. The only slightly negative genetic correlation was found between head and horn length (−0.05), where SE was 0.06 and, therefore, the genetic correlation was not different from zero. The highest genetically correlated traits were head length and eyes (0.76). The phenotypic correlations were lower than the genetic ones, ranging from 0.09 (horn length - head length, horn length - eyes) to 0.54 (head nobility - head length).

**TABLE 4:** Estimated genetic correlations ± standard errors (above diagonal) and phenotypic correlations (bellow diagonal) for scored autochthonous traits from Group 2.

Trait	Head length	Head nobility	Eyes	Horn base circumference	Horn length	Horns direction
Head length	—	0.59 ± 0.04	0.76 ± 0.04	0.38 ± 0.05	−0.05 ± 0.06	0.10 ± 0.07
Head nobility	0.54	—	0.74 ± 0.04	0.67 ± 0.04	0.31 ± 0.06	0.45 ± 0.07
Eyes	0.42	0.47		0.29 ± 0.04	0.18 ± 0.06	0.17 ± 0.07
Horn base circumference	0.20	0.41	0.23	—	0.60 ± 0.04	0.42 ± 0.05
Horn length	0.09	0.22	0.09	0.42	—	0.44 ± 0.05
Horns direction	0.14	0.32	0.23	0.32	0.37	—

The estimated phenotypic and genetic correlation between the neck and dewlap (Table 5) was positive and moderate (0.53). Animals with thin skin on the neck had a less expressed dewlap and vice versa, which was expected.

**TABLE 5:** Estimated genetic correlation ±standard error (above diagonal) and phenotypic correlation (bellow diagonal) for scored autochthonous traits from Group 3.

Trait	Neck	Dewlap
Neck	—	0.77 ± 0.05
Dewlap	0.53	—

The estimated genetic and phenotypic correlations between the scored traits describing the coat color and white stripes patterns (Table 6) were positive, very low one the hand or very high on the other. The coat color was weakly correlated with all traits describing white stripe patterns, whereas the back stripe, the rear legs stripe, and the front legs stripe were highly correlated with each other. Likewise, the genetic correlation between the coat color and the front legs stripe (0.02) had a SE of 0.03 and, therefore, was not different from zero. First parity Cika cows with a wider white back stripe had wider white strips on the rear and front legs. Cows with a wider white stripe on the rear legs had a wider white stripe on the front legs as well.

**TABLE 6:** Estimated genetic correlations ± standard errors (above diagonal) and phenotypic correlations (bellow diagonal) for scored autochthonous traits from Group 4.

Trait	Coat color	Back stripe	Rear legs stripe	Front legs stripe
Coat color	—	0.10 ± 0.02	0.06 ± 0.02	0.02 ± 0.03
Back stripe	0.07	—	0.97 ± 0.008	0.91 ± 0.02
Rear legs stripe	0.06	0.82	—	0.95 ± 0.01
Front legs stripe	0.01	0.75	0.80	—

The majority of the estimated correlations among the body frame traits (Table 7) were low to moderate. The lowest genetic correlation was found between rump angle and hock quality (−0.03), while the highest was between heel height and foot angle (0.89). However, the genetic correlation between rump angle and hock quality (−0.03) had a SE of 0.06 and was not different from zero. The highest phenotypic correlation (0.62) was also found between heel height and foot angle, whereas the lowest phenotypic correlation (−0.001) was between rear leg set and rump angle. Animals with a low foot angle had low heel height, while animals with a steep foot angle had high heel height. Moderate genetic correlations were estimated between the composite trait body frame and heel height as well as with foot angle (both 0.58). The composite trait body frame had the highest negative correlation with rear leg set (−0.59). Cows with high scores for the composite trait of body frame had relatively steep foot angles and high heel height, and a steep rear leg set. Špehar et al. (2012) estimated a similar genetic correlation between rear leg set and heel height (−0.33) in Slovenian Brown Swiss cows. Němcová et al. (2011) estimated lower genetic and the same phenotypic correlation between rump angle and heel height (−0.06; −0.04) in Czech Holstein-Friesian cows.

**TABLE 7:** Estimated genetic correlations ± standard errors (above diagonal) and phenotypic correlations (bellow diagonal) for scored and composite body frame traits from Group 5.

Trait	Top line	Rump angle	Rear leg set	Hock quality	Foot angle	Heel height	Body frame – composite trait
Top line		0.49 ± 0.07	−0.30 ± 0.15	0.23 ± 0.08	0.10 ± 0.13	−0.06 ± 0.15	0.31 ± 0.10
Rump angle	0.25	—	0.12 ± 0.11	−0.03 ± 0.06	0.09 ± 0.09	−0.12 ± 0.10	−0.40 ± 0.07
Rear legs set	−0.06	−0.01		−0.10 ± 0.05	−0.42 ± 0.10	−0.24 ± 0.12	−0.59 ± 0.09
Hock quality	0.07	−0.05	0.05	—	−0.12 ± 0.10	−0.40 ± 0.10	0.27 ± 0.07
Foot angle	0.07	−0.03	−0.26	−0.04	—	0.89 ± 0.05	0.58 ± 0.10
Heel height	0.02	−0.04	−0.14	−0.12	0.62	—	0.58 ± 0.09
Body frame – composite trait	0.23	−0.13	−0.35	0.12	0.59	0.54	—

The estimated phenotypic correlations between udder traits (Table 8) were moderate and positive (up to 0.79 between fore udder attachment and the composite udder trait), and weak to moderate negative (up to −0.39 for front teat length and udder depth). Some estimated genetic correlations were high and positive, with the highest correlation between udder depth and the composite trait “udder” (0.94). The others were moderate and negative (e.g. −0.74 between udder depth and front teat length). The lowest genetic correlation was estimated between front teat length and fore udder attachment (−0.45). A functional udder should be extended under the abdomen, well attached, with thin and short teats.

**TABLE 8:** Estimated genetic correlations ± standard errors (above diagonal) and phenotypic correlations (bellow diagonal) for scored udder traits and the composite trait udder from Group 6.

Trait	Fore udder attachment	Udder depth	Teat thickness	Front teat length	Udder – composite trait
Fore udder attachment	—	0.78 ± 0.06	−0.52 ± 0.10	-0.45 ± 0.10	0.89 ± 0.04
Udder depth	0.42	—	−0.71 ± 0.07	−0.74 ± 0.05	0.94 ± 0.03
Teat thickness	−0.09	−0.36	—	0.78 ± 0.04	−0.62 ± 0.09
Front teat length	−0.19	−0.39	0.59	—	−0.64 ± 0.08
Udder – composite trait	0.79	0.56	−0.22	−0.35	—

Fore udder attachment and udder depth were relatively strongly correlated (0.78), as well as front teat length and teat thickness (0.78). Cows with a genetic predisposition for weakly attached udders usually have deeper udders as well. Němcová et al. (2011) estimated higher phenotypic and lower genetic correlations between fore udder attachment and udder depth (0.44; 0.75) in Czech Holstein-Friesian cows. Mazza et al. (2014) estimated lower phenotypic and genetic correlations between fore udder attachment and the composite trait “udder” (0.68; 0.78) in Rendena cows.

### Genetic Trends

**FIGURE 1:**
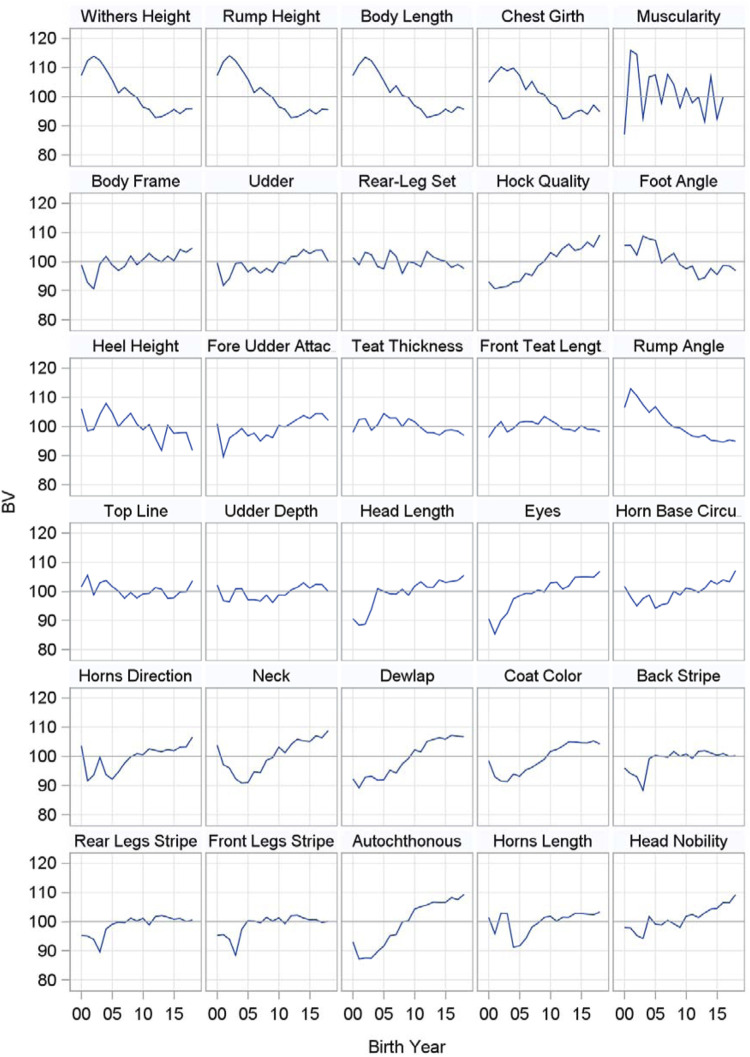
Genetic trends for type traits in first parity Cika cows born in the years 2000–2019.

All 26 individual type traits as well as the four composite traits were already introduced in the genetic evaluation of first parity Cika cows in the year 2016. The composite traits, which include all individual traits, are widely used. There is a plan to decrease the number of traits in the future since some of the traits within each group are highly correlated. Nevertheless, Figure 1 includes the genetic trends of all 26 individual type traits as well as the four composite traits for first parity Cika cows born from 2000 to 2019. The genetic trends of all four measured body frame traits are negative, which is desirable and expected since the breeders prefer Cika animals with a smaller body frame that were not admixed with Pinzgauer in the past. Moreover, potential Cika sires are genotyped each year to select those from the group suitable for artificial insemination that do not have genes of Pinzgauer or other foreign breeds. The autochthonous characteristics of the cows are represented by 12 individual traits divided into traits of the head, neck and coat color as well as coat color patterns. The genetic trends for the traits of the head are all positive. First parity Cika cows born in the last years had shorter and finer heads with larger eyes and thinner, shorter horns with a more forward direction compared to Cika cows born before 2010, which is in accordance with the breeding goals of the breeding program. A very similar situation is reflected by the positive genetic trends of the neck and dewlap, which became finer in younger cows. Likewise, heavier necks and dewlaps are typical for Cika cows that were admixed with Pinzgauer in the past. According to the genetic trends for coat color and its patterns, the coat color is becoming brighter, while white stripes on the back as well as on the rear and front legs have not changed very much in animals born during the last years. Among the scored body frame traits, only hock quality had a positive genetic trend, while rump angle, foot angle and heel height had negative genetic trends. Consequently, cows born in the last years have more sloping rumps, as well as cleaner and drier hocks with steeper angles of the foot and taller heels. In the past, a lot of cows had high pins and hocks with a lot of fluid, which was very undesirable for the breeders, i.e. the genetic trends are in accordance with the breeding goals now. Among the scored udder traits, only fore udder attachment and udder depth had slightly positive trends, which means that cows have more strongly attached and shallower udders than in the past. Finally, three (autochthonous characteristics, body frame, udder) of four composite traits had positive genetic trends. All in all, first parity Cika cows born in the last years showed more typical autochthonous type traits of purebred Cika cows, which distinguished them from Cika cows admixed with Pinzgauer in the past. Likewise, first parity Cika cows nowadays have more excellent body frames and udders.

## Conclusion

Comparing the estimated heritabilities for the majority of included type traits with heritabilities from the literature, it was found that they were within the expected range and similar to those reported for other breeds. Among the measured body frame traits, the highest heritability was estimated for rump height. The heritabilites of scored body frame traits in Cika were similar to those of other breeds as well. In the scoring form for Cika, only four scored udder traits are included (fore udder attachment, udder depth, teat thickness, front teat length). Nevertheless, their heritabilities were very similar to those reported for cows of dairy breeds. The estimated heritabilities of the scored autochthonous traits, which describe the breed characteristics, were moderate to high. This suggests the possibility to breed first parity Cika cows with unique type traits known for autochthonous Cika cattle. Unfortunately, according to the best of our knowledge, no autochthonous type traits like those included in the analysis have been investigated before. Consequently, comparison with the literature was not possible.

All above estimated genetic parameters are already used for breeding value prediction in the Cika cattle population. According to the genetic trends, first parity Cika cows born in the last years showed more typical autochthonous type traits of purebred Cika cows, which distinguished them from Cika cows admixed with Pinzgauer in the past. Likewise, first parity Cika cows nowadays have more excellent body frames and udders.

## Data Availability

The raw data supporting the conclusions of this article will be made available by the authors, without undue reservation.
